# Principles and Guidelines for In-Line Viscometry in Cereal Extrusion

**DOI:** 10.3390/polym14122316

**Published:** 2022-06-08

**Authors:** Elia Dalle Fratte, Dagmar R. D’hooge, Mia Eeckhout, Ludwig Cardon

**Affiliations:** 1 Department of Food Technology, Safety and Health, Faculty of Bioscience Engineering, Ghent University, Valentin Vaerwyckweg 1, 9000 Ghent, Belgium; elia.dallefratte@ugent.be; 2 Laboratory for Chemical Technology (LCT), Department of Materials, Textiles and Chemical Engineering, Ghent University, Technologiepark 125, Zwijnaarde, 9052 Ghent, Belgium; dagmar.dhooge@ugent.be; 3 Centre for Textile Science and Engineering (CTSE), Department of Materials, Textiles and Chemical Engineering, Ghent University, Technologiepark 70A, Zwijnaarde, 9052 Ghent, Belgium; 4 Centre for Polymer and Material Technologies (CPMT), Department of Materials, Textiles and Chemical Engineering, Ghent University, Technologiepark 130, Zwijnaarde, 9052 Ghent, Belgium; ludwig.cardon@ugent.be

**Keywords:** slit dies, poiseuille flow, expansion, starch, food processing

## Abstract

In the food industry, extrusion cooking finds numerous applications thanks to its high productivity and nutrient retention. More specifically, cereal extrusion, e.g., for savory snacks and breakfast products has an important market share. For such applications, rheology, which addresses viscous and elastic contributions, plays an important role in developing, optimizing, and controlling the extrusion manufacturing technique. In this context, conventional off-line rheometers are not ideal for providing data, as the goal is to replicate the exact thermomechanical history to which the food is subjected in the extrusion process. Hence, to achieve reliable analyses, in-line viscometers that have mostly been tested using oil-based polymers were introduced. Biopolymers (e.g., starch), however, are highly sensitive to both heat and mechanical degradation, and the viscometer design has to be adapted accordingly to produce an accurate measurement. Alongside a discussion of the different designs available, this review will address the most common methodologies for measuring the steady shear viscosity, extensional viscosity, and the first normal stress difference for food applications, providing researchers in the biopolymer and food engineering fields with a general introduction to this emerging topic.

## 1. Introduction

In the food industry, extrusion cooking is often preferred over other food-processing techniques because it is a continuous process with high productivity and significant nutrient retention [1]. The process has found numerous applications, as witnessed by the increasing amounts of ready-to-eat cereals, salty and sweet snacks, co-extruded snacks, indirect expanded products, croutons for soups and salads, as well as by an expansion of the array of dry pet foods and fish foods, textured meat-like materials from defatted high-protein flours, 3D-printed food, nutritious precooked food mixtures for infant feeding, and confectionery products [2,3,4,5,6,7,8,9].

Although the applications are many, the field of cereal extrusion represents the biggest, in terms of market value, and the most important, in terms of future growth. The market value of cereal extrusion alone is estimated to represent approximately USD 63 billion of the total USD 73 billion, and it is expected to grow at a compound annual growth rate (CAGR) of 4–5% in the period of 2022–2032 [10]. Industry ARC [11] reported in its market analysis that the category of savory snacks is forecasted to be the fastest growing segment and is set to register the highest CAGR during the period of 2020–2025.

Most cereals contain a large amount of starch. In its natural form, starch is unsuited for human consumption. To make it digestible and acceptable, it must be cooked. Cooking or the gelatinization of starch in the traditional cereal process is controlled by time, temperature, and the availability or presence of water. In the extrusion cooking process, shear is a fourth dimension that impacts product quality. The operational complexity of the process, however, remains one of the biggest challenges preventing further growth [12].

In this context, rheology is extremely valuable for both optimizing and controlling the process outcome, as the rheological properties are the driving factors of the flow behavior and the expansion phenomena. However, measuring the rheology for food products under extrusion conditions is far from trivial, even with standard off-line rheometers. In contrast, for oil-based polymer engineering and process design, in-line (directly in the process) and on-line (in a branch of the process) procedures are more developed [13]. Processes involving biopolymers are, however, more sensitive to structural degradation due to a highly complex and less understood dependency on heat and shear forces. Hence, the classical slit-die designs, which are widely employed in oil-based polymer applications, often produce unreliable data [14,15,16,17,18] and should be further adapted to enable well-defined food processing.

The main scope of this review is to present the most relevant (adapted) slit-die viscometers (SDVs) for the measurement of the rheological properties of extruded (food) products. The aim is to ease the development of new SDVs for thermo- and mechanical-sensitive materials in order to enhance the quality and consistency of future data recording in the food and bioengineering science fields, while taking starch as reference biopolymer.

The review will focus mostly on starch, for two important reasons. Firstly, there is an increasing interest in measuring the rheological data of extruded starch for both the production of bio-films and for the modeling of the expansion phenomena of extruded expanded products such as cereals and ready-to-eat snacks. Secondly, in the food industry, slit-dies have mostly been used for starch-based products. Hence, the most available rheological data are on starch.

From a technical point of view, this review aims to present the history of slit-die designs by addressing the pros and the cons. The main focus is on the expansion phenomena as understood from a rheological angle, the most important (in-line) viscometric techniques, and possible further developments.

## 2. Rheological Challenges

In the food extrusion process, the raw material is subjected to shear forces according to the feed rate, screw geometry, screw rotation speed, and processing temperature. Due to these forces, the material melts and flows as a highly viscoelastic fluid towards the die, which is a special unit mounted at the end of the extruder to obtain a well-defined melt flow exit. At the die exit, the pressure drops to atmospheric pressure, which causes, in the case of starchy products containing low amounts of water, a rapid expansion due to sudden water vaporization and die swell [19,20,21,22]. At sufficiently high temperatures, this expansion phenomenon eventually gives the product its usual airy structure, which is responsible for the product’s crispy texture and final shape. For this reason, understanding the expansion phenomena is considered paramount for controlling, optimizing, and developing expanded products.

This expansion is, from a fundamental point of view, a complex phenomenon occurring in a short time interval. It involves the succession of dynamic steps such as bubble nucleation, bubble growth, coalescence, local shrinkage, and, finally, setting. Notably, Moraru and Kokini [19], in their review, highlight the importance of the die pressure drop in the formation of vapor nuclei. More specifically, nucleation starts if the vapor pressure of the fluid is approached. Consequently, nucleation is determined by the pressure profile within the die, which in turn depends on the viscosity of the melt and the die geometry [23,24]. Moreover, for an optimal expansion, Bouvier and Campanella [25] state that small die diameters with short lengths are favorable.

Once formed, the nuclei can grow bigger. According to the “cell model” of Amon and Denson [26], the growth occurs due to the diffusion of dissolved gas from the matrix into the bubble. Such diffusion requires that the pressure difference between the inside and the outside of the bubble overcomes the resistant effect due to both the surface tension and the viscous forces. Shear viscosity should be low enough to promote bubble growth, but high enough to prevent bubble collapse and coalescence [19,27,28,29]. The same conclusion for the effect of viscosity on expansion has been reported for the production of foamed polymers [30,31].

An extra complexity arises due to the role of water in food preparation. As water evaporates outside of the die, the extrudate temperature and moisture content simultaneously decrease. Due to the moisture decrease, the glass transition temperature ($T_{g}$) increases [20,32]. Eventually, as the extrudate melt temperature approaches $T_{g}$, the viscosity and storage modulus drastically increase, the bubble growth stops, expansion ceases, and the structure consolidates. According to Fan et al. [33], the structural consolidation of starch-based extrudates occurs at a temperature ($T_{set}$) approximately 30 °C higher than $T_{g}$. Horvat and Schuchmann [34] performed a similar analysis on corn grids, reporting that the structural consolidation occurs at 45 °C above $T_{g}$. In any case, it can be concluded that if $T_{set}$ is higher than 100 °C (e.g., at low moisture content), the vapor bubbles swell and the structure is set before the possible collapse of the bubbles, which results in a higher expansion. Conversely, if $T_{set}$ is smaller than 100 °C (e.g., at high moisture content), the bubbles may shrink if the product temperature drops to below 100 °C due to vapor condensation.

Shear viscosity alone, however, is not enough to completely characterize the expansion phenomena. Because bubble growth is governed by the extension of the surrounding matrix, the extensional viscosity is considered to be the dominant strain mode in the last stages of expansion [20,35]. Therefore, measuring the viscous as well as the elastic behavior of the plasticized starch matrices is of great interest for understanding, controlling, and predicting the expansion of extruded melts.

It should be stressed that in more conventional oil-based polymer processing operations, such as polyolefin film blowing and foaming, it has been indicated that the polymer melt should exhibit an elongational viscosity that is high enough to withstand the stretching forces during expansion to avoid film rupture and coalescence [36]. This explains the development of many viscoelastic constitutive models for conventional polymer flow. However, in food engineering, less developed models are typically used due to the complexity of the system and computational power constraints. More specifically, the current models are not ideal for a very sensitive material such as food matter that can strongly degrade along the extrusion process due to thermomechanical forces.

In a more general context, it can be claimed that the flow behavior of starch polymer melts is non-trivial because of several features, which complicates the rheometric analysis and flow model development. Firstly, while water acts as a destructuring agent and as an efficient plasticizer during processing, most rheometers cannot preserve the water content, which makes conventional rheological methods often impossible to apply. Secondly, in contrast to synthetic polymer melts (e.g., polyethylene), biopolymer starches are very susceptible to molecular degradation due to high thermomechanical stresses [37,38]. This degradation induces changes in the rheological properties that are difficult to follow and replicate in offline devices. Thirdly, the viscosity of a starch polymer melt, especially that of the amylopectin fraction, is much higher than the viscosity of most synthetic polymers, which also makes the rheological characterization difficult [39].

Taking into consideration the above-mentioned limits and challenges, the most practical way to measure the rheological properties of (bio)polymers under the closest extrusion-like conditions is through in-line determination [16,40,41,42,43,44,45]. Here, the focus should ideally be both on viscous and elastic contributions, as highlighted in the next section.

## 3. In-Line Viscometric Measurement

### 3.1. Main Principles for Shear Viscosity

**Figure 1:**
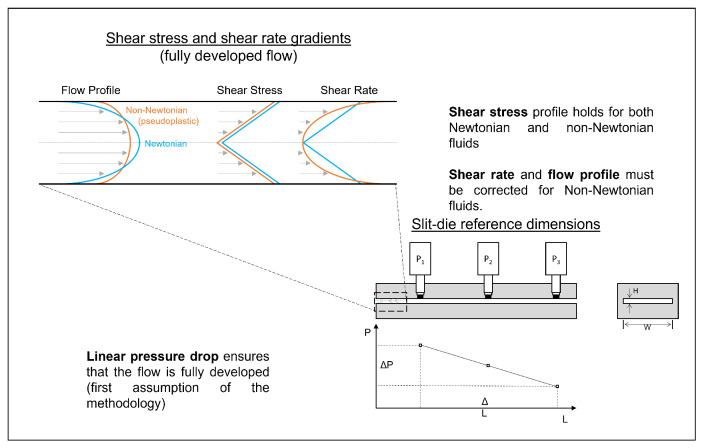
Shear rate determination requires special data treatment in the case of a non-Newtonian fluid. The Weissenberg–Rabinowitsch–Mooney correction is applied to the apparent shear rate to obtain the wall shear rate (Equation (4)).

In-line measurement of the rheological properties of molten extrudates can be achieved for moderate shear rates by coupling a laboratory-size single or twin-screw extruder with a capillary rheometer or a slit-die viscometer (SDV). However, for non-Newtonian fluids such as molten starch and most biopolymers in general, SDVs are globally preferred. This is because the pressure variation is measured directly inside the flow channel without interfering with the flow [43], as shown in Figure 1. Consequently, the Bagley pressure loss and correction, which is required for capillary viscometers, can be neglected [17].

To measure the rheological properties using an SDV, different shear rates must be generated within the slit channel. These different shear rates can be achieved by either varying the rotation speed or frequency [15,46,47,48,49,50,51,52,53] or by varying the feed rate [54,55,56,57,58,59,60,61,62,63]. However, both approaches interfere with the process, making the molten material subject to different thermomechanical treatments, which can in turn highly affect the rheological properties of the melt [14,15,16]. Therefore, although varying the screw speed or the feed rate seems to be a reasonable way of obtaining viscosity data from an SDV, the results must be carefully interpreted.

For twin-screw extruders, Van Lengerich [64] suggested that this issue may be addressed by controlling the specific feeding load (SFL), which is defined as the ratio between feed rate and screw speed. At constant SFL, the degree of filling and the specific mechanical energy in the extruder can be claimed more as constant, and the thermomechanical history is consequently changed, likely to a limited extent. This approach, however, leads to a very long experimental procedure, as proven by Li et al. [17].

The steady shear viscosity ($\eta_{sh}$) is the most common rheological property measured with an SDV, whose principle is shown in Figure 1. Its extrapolation from the flow data relies on a series of flush-mounted pressure transducers that measure the pressure directly inside the flow channel without any disturbance of the flow. One of the recommendations is a fully developed flow; hence, at least three pressure transducers should be used to assess the linearity of the pressure drop along the channel (see P1, P2, and P3 in Figure 1). Together with a fully developed flow, it is ideal to comply with the following constraints [16]: (i) isothermal conditions; (ii) negligible viscous heat dissipation; (iii) no-slip at the walls; (iv) laminar flow regime; (v) negligible end effects; (vi) no chemical or physical modifications, interactions, or changes in state along the device, which thus implies that the shear history in the device can be ignored. $\eta_{sh}$ can then be calculated according to Equation (1):

(1) $$\eta_{sh}=\frac{\tau_{w}}{{\dot{\gamma }}_{w}}$$

where ($\tau_{w}$) and (${\dot{\gamma }}_{w}$) represent the wall shear stress in Pa and the wall shear rate in $s^{-1}$, respectively. $\tau_{w}$ is calculated from the pressure drop (ΔP) along the length (*L*) of the slit rheometer and holds for both Newtonian and non-Newtonian fluids, as shown at the top left of Figure 1, and mathematically grasped by the following equation:

(2) $$\tau_{w}=\frac{\Delta PH}{2L}$$

${\dot{\gamma }}_{w}$ depends on the velocity profile within the channel, having a height of *H*, which differs between Newtonian and non-Newtonian fluids, as shown in Figure 1. For Newtonian fluids, ${\dot{\gamma }}_{w}$ is calculated from the volumetric flow rate through the slit channel (Equation (3)). For non-Newtonian fluids, this Newtonian shear rate is denoted as an apparent shear rate (${\dot{\gamma }}_{a}$), and its product with the Weissenberg–Rabinowitsch–Mooney correction (Equation (4)) is needed.

(3) $${\dot{\gamma }}_{w,Newtonian}=\frac{6Q}{WH^{2}}$$

(4) $${\dot{\gamma }}_{w}=\frac{{\dot{\gamma }}_{a}}{3}\left(2+\frac{\delta (\ln{\dot{\gamma }}_{a})}{\delta (\ln\tau_{w})}\right)$$

In Equations (3) and (4), *Q* is the volumetric flow rate (m${}^{3}$ s${}^{-1}$). For power-law fluids, the derivative $\frac{\delta (\ln{\dot{\gamma }}_{a})}{\delta (\ln\tau_{w})}$ in Equation (5) is constant and equal to 1/n, where *n* is the power-law index. This index is also a key parameter for the viscosity dependency of the shear rate:

(5) $$\eta_{sh}\left(\dot{\gamma }\right)=K{\dot{\gamma }}^{n-1}$$

in which *n* ranges between 0 and 1, and *K* is the consistency factor.

Typical values for *n* are reported in Table 1. The wide range in the reported values is due to the dependency of *n* on the temperature and moisture content of the extruded melt. It should be noted that some raw starch materials display shear thickening behavior [65,66,67]. However, for gelatinized (i.e. temperature-treated starch), shear thinning is expected under extrusion conditions (see Table 1) [68]. Furthermore, shear thinning has also been reported for wheat flour [59,69], corn [57,70], corn with different amylose/amylopectin ratios [71], corn meal [41], corn grids [17,72], and potato powder [57].

### 3.2. Technical Implementations and Modifications

**Figure 2:**
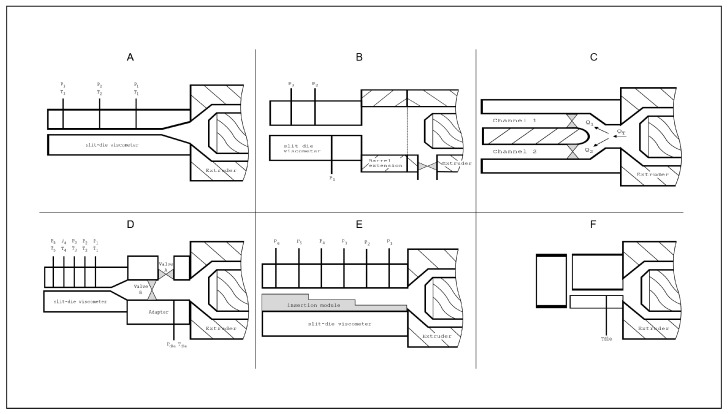
Slit-die viscometer designs. The dimensions are not representative of the original designs. The images solely aim to clarify the design components and construction. (**A**) Standard slit-die design. (**B**) Pre-valve design [82]. The valve—at the screw end—controls the flow within the slit channel. (**C**) Twin-channel design [81]. The flow within each channel is controlled by a dedicated valve. (**D**) Two-valve design [17]. One valve controls the flow within the measuring channel, while the other insures a constant back pressure. (**E**) Inner module design [43]. The interchangeable module allows for the control of the flow as well as the back pressure. (**F**) Dual orifice capillary die [83].

The technical implementation of the die concept can be realized in various manners (thus, beyond Figure 1; repeated in Figure 2A), as concisely explained in the present subsection. For a detailed description, the reader is referred to the work of Xie et al. [84].

To address the issue of generating different shear rates without interfering with the thermomechanical history, Padmanabhan and Bhattacharya [82] introduced the concept of using a side-stream valve. Later on, this concept was applied in a series of studies to characterize the rheological properties of corn meal [41,72,80,85,86]. In this design, as shown in Figure 2B, a side-stream valve is placed near the exit of a flood-fed single-screw extruder that is run at fixed screw speeds. By adjusting the opening of the side-stream valve, the flow rate through the slit die is controlled, and the shear rate varies. However, the rheological data obtained using this technique can be significantly different from those obtained by varying the screw speed, which, under some conditions, yields n<0 [82].

For completeness, it must be mentioned that a similar concept has been applied involving a twin-screw extruder [28,87]. However, with this design type applied on either single- or twin-screw extruders, no opening of the SDV channel can be achieved for an enlarged opening of the side-stream valve, in view of increasing the flow restriction to the SDV. Hence, the thermomechanical history would be affected. One always induces a pressure drop at the die entrance, which results in changes in the thermomechanical history of the melt [17,84]. The entrance pressure could be increased by increasing the feed rate; however, this would again interfere with the thermomechanical history of the melt.

Alternatively, Vergnes et al. [81] designed the so-called “Rheopac”, as shown in Figure 2C. This is an in-line viscometer based on the principles of Springer et al. [88] and used in the rheological studies of starch polymer melts [35,79,89,90,91,92]. This viscometer achieves a variation in the shear rate without interfering with the thermomechanical history, by using two geometrically identical channels—one for the measurements and the other for deviations of the flow. Each channel is provided with a valve to partially obstruct the flow section. A change in flow rate to the measurement channel can therefore be balanced by a variation of the flow in the second channel, hence maintaining a constant entrance pressure. Another advantage of this viscometer is that the measurements can be performed more swiftly than with a single-channel slit-die since no waiting time for the stabilization of the melt flow is required after adjusting the flow [81].

The approach of Vergnes et al. [81] was recently expanded by Teixeira et al. [93], who generalized the flow analysis to accommodate different channel lengths and cross-sections. The necessity for an accurate measurement of the flow rate in each channel is, however, a disadvantage of both the twin and double-channel rheometers.

Li et al. [17] introduced a different concept to overcome the drawbacks of implementing a side-stream valve (i.e., lack of back-pressure control, hence, different thermomechanical history). This was accomplished while maintaining the advantages of a double-channel viscometer (i.e., constant thermomechanical history). They fitted an adapter between the SDV and the extruder, as shown in Figure 2D, allowing for the diversion of flows. Both the flow restriction towards the bypass channel and the one towards the SDV could be controlled by a dedicated valve. By adjusting the openings of the two valves, the flow rate through the SDV is varied, allowing for different shear rates while the back pressure is maintained constant. Instead of calculating the relationship between the two valve openings, in the case of the Rheopac, a pressure transducer was mounted just before the two valves. The pressure reading was used to monitor and maintain the entrance pressure ($P_{0}$) at a constant value while the valve openings were adjusted, assuring a constant thermomechanical history. Another advantage of this design compared to the Rheopac is that the operation of the two valves is independent of the power-law index [17].

More recently, Horvat et al. [43] developed a single-channel SDV with an exchangeable inner geometry, as shown in Figure 2E. Later on, this design was used to characterize the behavior of rice starch fortified with pea proteins [2] and maize starch [29,45]. Several advantages have been put forward. Firstly, the modularity of this design gives the possibility to control the back pressure by adapting either the length or the height of the flow channel. Secondly, a large shear rate range could be covered (1–2000 s${}^{-1}$) by changing the slit height. Thirdly, a multiple-step geometry enables the measurement of the viscosity of at least three shear rates during one experiment, thus assuring a flow curve at the constant thermomechanical history of the processed material. The main drawback of this design is that all viscosity data will result from two pressure readings in the case when the multi-step module is used. However, at least three pressure readings should be taken to ensure a fully developed flow. Furthermore, in case a single-step module is used, changing the shear rates at constant back pressure will require a change of the inner module, which is a time-consuming procedure. Moreover, it can be expected that higher back pressures will result due to the changing die pathway.

Differently from the previous designs, Drozdek and Faller [83] used a dual orifice capillary die attached to a twin-screw extruder, as shown in Figure 2F, in order to determine the viscosity of starch polymer melts, assuming a power-law model (Equation (5)). This die enables two flow rates to be measured for one extruder condition. It has been claimed that this design provides a more accurate and time-efficient determination of *n* than any other in-line method. However, due to the geometry restrictions, pressure readings were not possible either at the bifurcation of the flow or along the channels. Hence, this rheometer was not suitable for the determination of *K*.

### 3.3. Beyond Steady Shear Viscosity

As discussed before, the melt shear viscous behavior ($\eta_{sh}$) is only partially responsible for characterizing the expansion, and ideally, the elastic component should also be taken into consideration. The elastic behavior of a starch polymer melt can be correlated with the first normal stress difference ($N_{1}$) and the planar extensional viscosity ($\eta_{e}$), both measurable with an SDV. However, compared to $\eta_{sh}$, both $N_{1}$ and $\eta_{e}$ have received minimal attention, likely due to the complexity of the rheometric treatment and the practical difficulties in the measurement, which is even more considerable for biopolymers and with even less focus on very dedicated rheological analyses. Nevertheless, $N_{1}$ and $\eta_{e}$ are often far more sensitive than $\eta_{sh}$ to the changes or differences in melt microstructure and to the addition of ingredients [72,94,95,96]; hence, their measurement remains essential.

Despite the above-mentioned difficulties, some results for $\eta_{e}$ are already available for starch products, including starchy recipes for ready-to-eat breakfast cereals using both slit-die [80,85] and conventional off-line rheometers [97,98]. It has been suggested that $\eta_{e}$ approximately follows a power-law of the elongational rate and is influenced by the moisture content and the temperature.

The importance of taking $\eta_{e}$ into account is reinforced by looking at the Trouton number, which is defined as the ratio of the $\eta_{e}$ to the $\eta_{sh}$. High Trouton numbers indicate that $\eta_{e}$ is greater than $\eta_{sh}$. Compared to the Trouton number of Newtonian fluids, which is equal to 4 [99], that of starch melts and synthetic polymers exhibiting long-chain branching is higher. For extruded corn meal, Bhattacharya et al. [85] reported, e.g., Trouton values in the range of 25–50, with lower values if the moisture and temperature are increased. Senouci and Smith [58] reported Trouton values between 75 and 200 for corn grits extruded at 120 °C, and higher than 1000 for potato flour. More recently, Núnez et al. [98] reported values between 250 and 2200 for a more complex, ready-to-eat formulation (70% oat flour, 13% rice flour, 17% minor components, among which are malt extract, sugar, vitamin, and salt).

#### 3.3.1. The First Normal Stress Difference

Using an SDV, $N_{1}$ can be estimated by either the exit pressure ($P_{e})$ method [23,100,101] or the hole pressure ($P_{h}$) method [102,103,104].

$P_{e}$ is related to the residual stress exerted by the fluid at the exit of the viscometer/die as the fluid flows into the atmosphere. The (conventional) exit pressure theory, as used for measuring $N_{1}$ of the polymer melt flow through a planar geometry, is based on the macroscopic momentum balance theory [105]. More specifically, assuming that a fully developed flow remains until the die exit, and that the inertial effect can be neglected due to a low Reynolds (Re) number, Han [23] and Davies et al. [106] derived the following analytic expression linking $P_{e}$ and $N_{1}$:

(6) $$N_{1}=P_{e}+P_{e}\frac{\delta \log P_{e}}{\delta \log\tau_{w}}$$

Because the experimental implementation of this method is difficult due to errors in the linear extrapolation of $P_{e}$, which sometimes even results in negative values [50,58,107], $P_{h}$ is generally preferred. For completeness, more recent simulations utilizing polypropylene have shown that the linear extrapolation of pressure values toward the die exit is suitable for determining $N_{1}$ despite the fact that these extrapolated $P_{e}$ values are characterized by a relative deviation of 25–40% [101].

**Figure 3:**
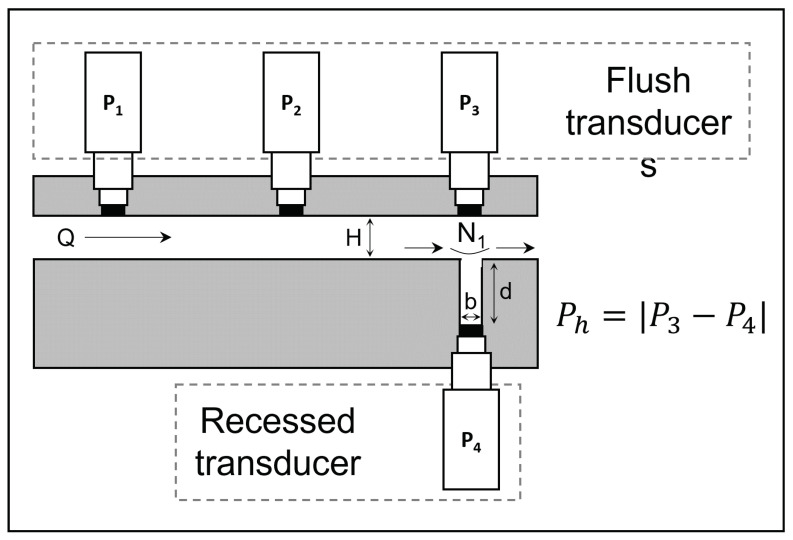
Representation of the position of the recessed transducer for the measurement of the first normal stress difference ($N_{1}$) based on the hole pressure method.

The $P_{h}$ method, as shown in Figure 3, uses the pressure difference between a flush and a recessed transducer located below a slot, machined along the width of an SDV that is perpendicular to the direction of the flow and fixed exactly opposite each other. For the determination of $N_{1}$, the Higashitani–Pritchard–Baird equations (HPB), based on the work of Higashitani and Pritchard [108] and later differentiated by Baird [109], can be used:

(7) $$N_{1}=2P_{h}\frac{\delta \ln P_{h}}{\delta \ln\tau_{w}}$$

Even though a number of the assumptions made during the derivation of Equation (7) were later proved to be invalid [110,111], Yao and Malkus [112] showed that the errors due to violated assumptions exactly canceled each other, and they concluded that the final equation to calculate $N_{1}$ from $P_{h}$ derived by Higashitani and Pritchard [108] should be representative. For Newtonian and inelastic liquids under laminar conditions, $P_{h}$ is zero. For elastic liquids, $P_{h}$ is always positive.

There are two critical aspects when using $P_{h}$. The first one is the dimension of the slot width (*w*) and slot depth (*d*). Interestingly, Teixeira et al. [44], who investigated the intrinsic source of variability of the method, found that $N_{1}$ stabilizes and becomes independent of the slot size for w≥1 mm. The second critical aspect is the absence of an absolute method to verify the accuracy of $N_{1}$. There is thus strictly no full certainty on the theoretical accuracy of $P_{h}$, and one can also encounter experimental deviations.

Furthermore, Lodge and De Vargas [113] summarized the experimental errors associated with the measurements of $P_{h}$. Besides the limitations associated with transducer sensitivity, errors arise if there is an appreciable misalignment between the two pressure transducers involved in the measurement, and if a small leakage of the polymer melt out of the slot unit occurs. Other possible sources of error are: (i) effects due to the slit-die geometry, (ii) viscous heating, (iii) variation of temperature during a series of measurements, (iv) thermal degradation of the material in the slot, and (v) wall slip [44]. Additionally, because $P_{h}$ represents a small difference between two high pressures, typically less than 2% of the measured pressure [44,113], the accuracy of the pressure measurements is essential to obtain reasonable data (otherwise, $P_{h}$ would be lost in experimental noise). It has also been suggested that the pressure transducers can be placed as far downstream as possible so that $P_{h}$ becomes the largest possible fraction of the measured pressure [44].

Despite the experimental difficulties associated with this method, researchers have investigated the validity of $P_{h}$, which remains the most promising methods for $N_{1}$. Lodge and De Vargas [113] and Seethamraju and Bhattacharya [72] used $P_{h}$ for low-density polyethylene (LDPE); they compared the results with those for off-line rheological measurements and found reasonable agreement. Padmanabhan and Bhattacharya [114], on the other hand, performed measurements of $P_{h}$ for two LDPEs of different average molar mass, but the values of $N_{1}$ they obtained did not agree well with those extrapolated from off-line rheometric data. More recently, Baird [115] reported, for four polymer melts, a reasonable agreement between $N_{1}$ values and those extrapolated from small-amplitude oscillatory shear data using the empirical Laun’s equation.

Finally, Teixeira et al. [44], who expanded the work of Baird [115], provided a quantitative analysis of intrinsic error sources, demonstrating that the acquisition of high-accuracy measurements is possible with the use of on-the-fly oversampling techniques.

#### 3.3.2. Planar Extensional Viscosity

$\eta_{e}$ describes the resistance of a fluid/melt to extensional flow. Any flow field involving a change in its cross-sectional area will be affected by this material property. Every extruded product is therefore subjected to such resistance. More recently, this has been further demonstrated by combined measurements [98,116].

Generating a controlled extensional flow for measuring $\eta_{e}$ is difficult in practice. Approximate analyses such as those provided by Cogswell [117] and Binding [118] allow for the assessment of $\eta_{e}$ from the entrance pressure drop ($\Delta P_{en}$). Such an estimation is not perfect, but it is valuable for at least ranking the fluids based on their extensional flow behavior.

Following the derivation of Cogswell, who assumed a power-law dependence of shear viscosity but a Newtonian extensional viscosity, the planar extensional viscosity and extensional rate can be calculated as follows:

(8) $$\eta_{e}=\frac{3{\left(n+1\right)}^{2}}{4}\frac{\Delta P_{en}^{2}}{\tau_{w}{\dot{\gamma }}_{a}}$$

and

(9) $${\dot{\epsilon }}_{e}=\frac{2}{3(n+1)}\frac{\tau_{w}{\dot{\gamma }}_{a}}{\Delta P_{en}}$$

in which $\Delta P_{en}$ is the entrance pressure drop.

If a power-law dependence of $\eta_{e}$ on ${\dot{\epsilon }}_{e}$ is assumed, which is generally expected [48,72,85,86,119], $\eta_{e}$ can be expressed as follows:

(10) $$\eta_{e}=S{\dot{\epsilon }}_{e}^{t-1}$$

where *t* is the power-law index, and *S* is the consistency coefficient.

A small value of *t* in Equation (10), which can be expected for corn meal [80], corresponds to the dominance of extensional flow over the shear component. Consequently, $\Delta P_{en}$ will be governed predominantly by $\eta_{e}$ rather than $\eta_{sh}$. Hence, $\eta_{e}$ is an important material function in designing extruders and dies that have food applications in mind.

## 4. Conclusions

Despite the fact that different techniques (off-line and on-line) have been employed to measure the rheological properties of starch-based melts in extrusion processes, in-line slit viscometry is the most preferred. The slit-die design, as imported from rheological studies on conventional oil-based polymers, has been adapted over the last years to fit the application. As starch-based biopolymers (and food material in general) are highly sensitive to thermomechanical stresses, one likely needs the use of more sophisticated designs that are capable of altering the flow within the slit channel without interfering with the thermomechanical history of the melt. This control is achieved by tailor-made slit-dies with the capability of monitoring and controlling the back pressure at the die entrance.

In the present work, the advantages and disadvantages of the SDVs have been provided. The main focus has been on the shear viscosity, but the technical implementation with regard to the first normal stress difference and the elongational viscosity have also been included.

It further follows that limited data recording has been performed for the food engineering field. With the further development of measurement tools, it can be expected that this scientific gap will be solved in the near future.

## Figures and Tables

**Table 1:** Typical values for the power-law index (*n*) reported for extruded starch-based products.

Author	Material	Method	*n* (Power Law Index)	
			min	max
Emin et al. [45]	Maize starch	Modular Design (Figure 2C)	0.375	0.395
Philipp et al. [2]	Rice starch + Pea proteins (0–30%)	Modular Design (Figure 2C)	0.229	0.394
Emin and Schuchmann [73]	Maize starch	Modular Design (Figure 2C)	0.325	0.395
Emin and Schuchmann [74]	Maize starch	Offline rheometer	0.375	0.395
Emin et al. [75]	Maize starch	Modular Design (Figure 2C)	0.329	0.52
Tajuddin et al. [76]	Waxy Maize starch	Offline rheometer	0.30	0.92
Chen and Ramaswamy [77]	Tapioca starch	Rotational viscometer	0.417	0.778
Willett et al. [78]	Waxy Maize starch	-	0.54	0.63
Della Valle et al. [79]	Maize starch (different ratio for amylose:amylopectin)	Rheopac (Figure 2C)	0.10	0.66
Li et al. [17]	Corn grids	Rheopac (Figure 2D)	0.30	0.49
Padmanabhan and Bhattacharya [80]	Corn meal	Rheopac (Figure 2B)	0.296	0.443
Vergnes et al. [81]	Maize starch	Rheopac (Figure 2C)	0.35	0.52
